# Atrioventricular Conduction Delay in Fetuses Exposed to Anti-SSA/Ro and Anti-SSB/La Antibodies: A Magnetocardiography Study

**DOI:** 10.1155/2012/432176

**Published:** 2012-12-20

**Authors:** Annette Wacker-Gußmann, Henrike Paulsen, Isabelle Kiefer-Schmidt, Joerg Henes, Jana Muenssinger, Magdalene Weiss, Rangmar Goelz, Hubert Preissl

**Affiliations:** ^1^Department of Neonatology, University Children's Hospital Tuebingen, 72076 Tuebingen, Germany; ^2^MEG and fMEG Center, University of Tuebingen, 72076 Tuebingen, Germany; ^3^Department of Obstetrics and Gynecology, University Hospital Tuebingen, 72076 Tuebingen, Germany; ^4^Centre for Interdisciplinary Clinical Immunology, Rheumatology and Autoinflammatory Diseases and Department of Internal Medicine II (Oncology, Hematology, Immunology, Rheumatology, Pulmonology), University Hospital Tuebingen, 72076 Tuebingen, Germany

## Abstract

*Background*. The presence of anti-SSA/Ro and anti-SSB/La antibodies during pregnancy is associated with fetal congenital heart block (CHB), which is primarily diagnosed through fetal echocardiography. Conclusive information about the complete electrophysiology of the fetal cardiac conducting system is still lacking. In addition to echocardiography, fetal magnetocardiography (fMCG) can be used. fMCG is the magnetic analogue of the fetal electrocardiogram (ECG). *Patients and Methods*. Forty-eight pregnant women were enrolled in an observational study; 16 of them tested positive for anti-SSA/Ro and anti-SSB/La antibodies. In addition to routine fetal echocardiography, fMCG was used. Fetal cardiac time intervals (fCTIs) were extracted from the magnetic recordings by predefined procedures. ECGs in the neonates of the study group were performed within the first month after delivery. *Results*. The PQ segment of the fCTI was significantly prolonged in the study group (*P* = 0.007), representing a delay of the electrical impulse in the atrioventricular (AV) node. Other fCTIs were within normal range. None of the anti-SSA/Ro and/or anti-SSB/La fetuses progressed to a more advanced heart block during pregnancy or after birth. *Conclusion*. The study identified a low-risk population within antibody positive mothers, where PQ segment prolongation is associated with a lack of progression of the disease.

## 1. Introduction

Fetal exposure to anti-SSA/Ro and anti-SSB/La antibodies is associated with the development of congenital heart block (CHB). The incidence is about 2% in primigravid mothers. The risk is five to tenfold higher in women who previously had an affected child with either CHB or a neonatal lupus rash [1, 2]. Fetuses with CHB carry high rates of mortality (20%) and morbidity (>60% of the surviving children require a permanent pacemaker in adulthood) [3, 4]. 

 Risk factors associated with a poor outcome are gestation <20 weeks, ventricular rate ≤50 bpm, hydrops, and impaired left ventricular function [4, 5]. In addition to the expected course of the disease, life-threatening cardiomyopathy is also found in the offspring in 10%–15% of cases and can occur in utero or postnatally [6]. 

 The pathogenesis of the disease is presumed to involve transplacental maternal IgG autoantibodies, which cause an atrioventricular (AV) delay. However, the first manifestation may also be sinus node dysfunction, atrial or ventricular ectopies or bundle branch blocks, but also junctional ectopic tachycardia or ventricular tachycardia [7]. Regarding the pathogenesis of these variable expressions of immune-mediated fetal cardiac disease, the spectrum is diverse. Alterations in the selective expression of calcium ion channels as well as accumulation of apoptotic cells were discussed [7–9]. 

 Current methods to monitor fetal heart function are based on echocardiography, Doppler, and tissue Doppler techniques, which provide indirectly through the mechanical assessment information of the fetal heart rhythm. Arrhythmias can be roughly classified, but details of the cardiac electrophysiology are incomplete. 

 Zhao and colleagues [10] reported underdetection in approximately 30% of cases with paroxysmal brief arrhythmias (junctional or ventricular tachycardia) associated with isoimmune CHBs using echocardiography. 

 To improve the complete electrophysiological assessment, further approaches to cardiac monitoring in these fetuses are needed. Fetal magnetocardiography (fMCG) is a new, noninvasive, and preclinical method, which can be used in addition to echocardiography. The groundwork for MCG analysis was built on different MCG devices primarily constructed for adults. Cardiac time intervals (CTIs) can be determined in an ECG-like fashion by recording magnetic fields generated by electric currents in the fetal heart. In addition, this method allows the detection of heart rhythm, heart rate trends, signal amplitudes, and unsuspected arrhythmias [11, 12]. 

 Therefore, the aim of our study was to evaluate whether fMCG can be used to detect *early* electrophysiological signs of atrioventricular delay in antibody-exposed fetuses. The primary endpoint of the study was the prolongation of atrioventricular conduction. 

## 2. Patients and Methods

### 2.1. Patient Population

Data collection for this controlled observational study at the fMEG Center Tübingen was completed in July 2012. Baseline characteristics of all 48 patients were evaluated with regard to medical history, previous pregnancy outcomes, and medication intake. 

Sixteen fetuses of pregnant women were included in the study group. These patients were measured up to four times. Therefore, each measurement of the study group was matched by gestational age to one of the control group.

 At study entry, all patients of the study group fulfilled the following inclusion criteria: presence of anti-SSA/Ro and/or anti-SSB/La antibodies tested by an enzyme linked immunosorbent assay (ELISA) and/or an immunofluorescence test, an immunodiffusion test and dot blots by a commercial laboratory. Rheumatologic disease was diagnosed by a rheumatologist. There was no limit concerning the duration of medication intake. Pregnancies >20 weeks of gestation with a normal heart beat and a structural normal heart were included.

 Healthy women with uncomplicated pregnancies and normally developing fetuses served as controls. Neonatal outcome including normal fetal heart rate was assessed by a pediatrician.

 Exclusion criteria for all neonates were chromosomal abnormalities, malformations, congenital infections, and/or acidosis at birth (umbilical artery cord gas pH < 7.0 or APGAR score after 5 minutes <5). 

 The study was approved by the ethics review board of the University Hospital Tübingen. Informed written consent was obtained from each subject.

### 2.2. Methods

At the beginning of the study, conventional echocardiography was performed on the study group to evaluate structural cardiac abnormalities, myocardial function, and fetal heart rate, in addition to the regular ultrasound check.

 fMCG measurements were also performed on the study group and on the control group. Each measurement was matched to one from a healthy fetus based on the gestational age (GA). fMCG analysis was conducted by three blinded observers. 

Prior to the beginning of each fMCG measurement, ultrasound was performed in all patients to check the fetal position and localise the fetal heart. Furthermore, cardiotocography (CTG) was performed over a 20-minute period to obtain complete information about the health of the fetus. 

### 2.3. Measurement Technique

fMCG is a noninvasive method for recording magnetic fields generated by the electric currents of the fetal heart [12]. It records magnetic fields generated by electrical currents in the fetal heart with highly sensitive sensors, so-called superconducting quantum interference devices (SQUID). SQUID sensors enable the display of fetal CTIs and provide detailed beat-to-beat analysis. The fMCG recordings were acquired using a 156-channel biomagnetic system (SARA system, VSM Med Tech Ltd. Port Coquitlam, Canada) for 15–45 minutes at a sampling rate of 1220.7 Hz. The data were analysed afterwards according to a recently implemented procedure for the fMEG-system: a bandpass filter was used between 1 and 100 Hz. The maternal MCG signal was detected and removed by signal space projection [13–15]. The fetal heart signal was detected, and the *R* peak was marked using the same technique. The marked fetal signals were averaged with a pre- and post-trigger-interval to extract the fMCG trace. 

The time points identified were used to calculate the duration of the CTIs as follows:

*P* wave = *P*_end_ − *P*_onset_, QRS complex = QRS_end_ − QRS_onset_, *T* wave = *T*_end_ − *T*_onset_. The QT interval was defined as QRS complex + ST segment + *T* wave. The PR interval was determined as *P* wave + PQ segment.

### 2.4. Laboratory Analysis

Anti-SSA/Ro and anti-SSB/La antibodies were detected using an ELISA test and/or an immunofluorescence test, an immunodiffusion test and dot blots. The ELISA test (Laboratory Seelig, Karlsruhe, Germany) is a very sensitive test and has a reference value <50 U/mL for anti-SSA/Ro and anti-SSB/La antibodies. The immunofluorescence test (Laboratory Klein, Tübingen, Germany) has a high specificity but less sensitivity. This titre information was available in positive and negative categories. Patients who tested positive for elevated levels of anti-SSA/Ro and anti-SSB/LA antibodies on the ELISA and/or by the immunofluorescence test, the immunodiffusion tests and dot blots were included in the study.

### 2.5. Statistical Analysis

Statistical analysis was performed using SPSS 20.0 (IBM) for Windows. All items were tested for a normal distribution using the Kolmogorov-Smirnov Test. As the data were normally distributed, the *t*-test was used for all CTIs. *P* < 0.01 was regarded as statistically significant. 

## 3. Results

### 3.1. Patient Population

#### 3.1.1. Study Group

 Sixteen mothers were included in the study group. The median age of the mothers with systemic lupus erythematosus (*n* = 11) or Sjögren's syndrome (*n* = 5) was 32 years (range 21–46 years). Anti-SSA/Ro antibodies were found in eleven patients (>3000 U/mL, *n* = 1, >225 U/mL, *n* = 6, <225 U/mL, *n* = 4). Anti-SSB/La antibodies yield in five patients (>700 U/mL, *n* = 1, >225 U/mL, *n* = 4). Five patients had both antibody types. Patients with detected antibodies had an obvious clinical disease. The maternal suppressive therapies in these 16 patients were high-dose prednisolone (*n* = 2), low-dose prednisolone (*n* = 10), hydroxychloroquine (*n* = 9), cyclosporine (*n* = 1), and azathioprine (*n* = 4). One patient completely refused therapy. Most of the patients received more than one medication.

 Sixteen fetuses were measured with a median gestational age of 31 weeks (range 24–38 weeks). The neonatal outcomes revealed eleven term newborns. Five neonates were premature (32–37 weeks GA). Fourteen neonates were healthy, whereas two fetuses showed a thrombopenia, but no further treatment was necessary. 

#### 3.1.2. Control Group

The median age of the 32 mothers in the control group was 32 years (range 23–40 years). All of these pregnant women were healthy. Pregnancy was not influenced by a previous disease. Thirty-two healthy fetuses were measured with a median GA of 31 weeks (range 24–38 weeks). One fetus was measured twice. The neonatal outcomes included 32 healthy fetuses without any abnormalities. All fetuses were born at term, except for one, who was born at 33 weeks gestation.

Baseline characteristics of the patients are shown in Table 1.

### 3.2. Fetal Cardiac Time Intervals

Cardiac time intervals were calculated in 16 patients of the study group and 32 patients of the control group. Altogether, 66 measurements in 48 patients were included in the complete analysis.

 Five patients in the study group were measured once, six patients were measured twice, four patients were measured three times, and one patient was measured four times with at least two weeks between consecutive measurements. Fetuses in the control group were measured once, except for one, who was measured twice. 

Therefore, each measurement was matched based on the gestational age of the fetus to a measurement from a healthy fetus. 

The average heart rates of both cohorts were 135 ± 10 beats per minute (bpm). The heart rates were within normal limits [16]. 

The cardiac time intervals for all patients are shown in Table 2. The PQ segment (isoelectric segment between the end of the *P* wave and the beginning of the QRS complex) was significantly prolonged (*P* = 0.007) in the study group compared to the control group. Other CTIs did not differ significantly. The T wave and QT interval yielded a low identification, especially in early gestational ages.

### 3.3. Postnatal Electrocardiograms

Thirteen postnatal electrocardiograms in the study group were analysed. All neonates had a normal sinus rhythm, and none had a congenital heart block. The median PR interval was in the normal range (100 ms) with reference to a mean heart rate (129 ± 13 bpm). The results were comparable with the widely used norm values of newborns reported by Park and Gunteroth (PR interval = 100–110 ms within 120–140 bpm) [17]. The median PQ segment and the P wave were within the normal range (50/50 ms). The prolongation of the PQ segment observed in the fetuses was not obvious in the neonatal ECG.

## 4. Discussion

The main finding in this study was that the PQ segment (PR interval—*P* wave), measured by fMCG, was significantly prolonged in the study group whereas all other cardiac time intervals were within normal range. 

 Van Leeuwen et al. [12] reported on PQ segment duration in healthy fetuses. Notably, this parameter was dependent on fetal heart rate and gestational age. Based on the careful matching of gestational age and no observed differences in heart rate, our results were not influenced by either effect. 

 For clinical decisions, PQ segment greater than the normal mean but within two standard deviations is not classified as first-degree AV block. The PQ segment prolongation might more reflect an IgG antibody effect in the development of atrioventricular node damage. 

 The molecular mechanisms leading to complete heart block are still unclear, but maternal antibody deposits were found in the heart of fetuses dying of congenital heart block and were thought to contribute to an inflammatory reaction that eventually induces fibrosis and calcification of the AV node [18].

 Boutjdir and colleagues [19] confirmed this in fetal cardiac preparations. Anti-SSA/Ro antibodies caused reversible blockade of L-type calcium channels. The authors proposed an initially and reversibly inhibition of inward calcium flux through L-type calcium channels. This effect can cause a delay or interruption of the atrioventricular conduction. First-degree congenital heart block in a fetus without progression suggests that L-type calcium channels can be dynamically altered in fetuses [7, 20, 21]. It is still unclear when the point of transition to irreversibility occurs. 

 Therefore the PQ segment prolongation in this study might reflect the early antibody effect, as these findings could not be confirmed by neonatal ECG. 

 Several limitations have to be considered, most of which are related to the small number of patients and the multiple measurements in the study.

 In conclusion, the morbidity and mortality associated with complete congenital heart block suggest the need for effective cardiac monitoring to avoid pre- and antenatal complications. This study population represents a “low-risk population” within antibody positive mothers, where PQ segment prolongation is associated with a lack of progression of the disease. The results might represent an early antibody effect. 

## Figures and Tables

**Table 1:** Baseline characteristics of the patients.

	Study group (*n* = 16 pts)	Control group (*n* = 32 pts)
Median age of mothers (years; range)	32 (21–46)	32 (23–40)
Height (meters; SD)	1.67 (±0.08)	1.68 (±0.07)
Body mass index before pregnancy	26 (±6)	24 (±4)
Body mass index during pregnancy	28 (±7)	27 (±4)
Prednisolone ≤10 mg (*n*)	10	0
Prednisolone >10 mg (*n*)	2	0
Dexamethasone (*n*)	0	0
Hydroxychloroquine (*n*)	9	0
Azathioprine (*n*)	4	0
Cyclosporine (*n*)	1	0
Male newborns (*n*)	8	19
Female newborns (*n*)	8	13
Mean birth weight (g; SD)	2902 (±510)	3489 (±550)
Mean birth length (cm; SD)	49 (±2)	51 (±2.5)

**Table 2:** Fetal cardiac time intervals.

Characteristics	Study group *n* = 33 measurements mean ± SD (ms)	Control group *n* = 33 measurements mean ± SD (ms)	Statistical significance (*t*-test)
Fetal heart rate (beats per minute)	135 ± 10	135 ± 10	ns
P wave	53 ± 15	58 ± 13	ns
PQ segment	56 ± 10	49 ± 10	*P* = 0.007
PR interval	109 ± 17	107 ± 15	ns
QRS complex	53 ± 7	52 ± 7	ns
T wave	168 ± 52*	140 ± 28^#^	ns
QT interval	261 ± 49*	232 ± 35^#^	ns

^∗^ *n* = 21 measurements, ^#^ *n* = 18 measurements.
